# Association between Shift Work and Neurocognitive Function among Firefighters in South Korea: A Prospective before–after Study

**DOI:** 10.3390/ijerph17134647

**Published:** 2020-06-28

**Authors:** Kyeongmin Kwak, Bong-Kyu Kim, Tae-Won Jang, Chang Sun Sim, Yeon-Soon Ahn, Kyeong-Sook Choi, Kyoung Sook Jeong

**Affiliations:** 1 Department of Occupational and Environmental Medicine, Korea University Ansan Hospital, Ansan 15355, Korea; ggm1981@snu.ac.kr; 2 Department of Environmental Sciences, Seoul National University Graduate School of Public Health, Seoul 08826, Korea; bkbk2@hanmail.net; 3 Department of Occupational and Environmental Medicine, Daewoo Hospital, Geoje 53317, Korea; 4 Department of Occupational and Environmental Medicine, Hanyang University College of Medicine, Seoul 04763, Korea; om1024@hanmail.net; 5 Department of Occupational and Environmental Medicine, Ulsan University Hospital, University of Ulsan College of Medicine, Ulsan 44033, Korea; zzz0202@naver.com; 6 Department of Preventive Medicine, Yonsei University Wonju College of Medicine, Wonju 26426, Korea; ysahn1203@yonsei.ac.kr; 7 Institute of Genomic Cohort, Yonsei University Wonju College of Medicine, Wonju 26426, Korea; 8 Department of Neuropsychiatry, Eulji University School of Medicine, Daejeon 34824, Korea; cksinj@eulji.ac.kr; 9 Department of Occupational and Environmental Medicine, Wonju Severance Christian Hospital, Wonju 26426, Korea

**Keywords:** firefighter, shift work, neurocognitive function, sleep deprivation, CNSVS

## Abstract

*Background*: Recent research indicates that shift work is associated with neurocognitive function. However, studies that examine the association between shift work and neurocognitive function in firefighters have not yet been performed. We examined the effect of shift work on neurocognitive function in firefighters by measuring and comparing neurocognitive function before and after night shift. *Methods*: 352 firefighters from eight fire stations in South Korea were included in this study. We performed neurocognitive function test using central nervous system vital signs (CNSVS) during daytime work and on the next day after night work. We performed paired *t*-tests to assess differences between neurocognitive function before and after night work. We also compared neurocognitive function in insomnia and depression. We used a general linear model to analyze the associations between shiftwork schedule and the changes in neurocognitive function. *Results*: The neurocognitive function significantly decreased in six domains (composite memory, verbal memory, visual memory, complex attention, psychomotor speed, and motor speed) as did the neurocognitive index on the next day after night work compared with during day work. These decreased domains were the same following night work regardless of the type of shift work. *Conclusion*: Night work in firefighters may cause neurocognitive decline.

## 1. Introduction

Firefighters in South Korea are responsible not only for fire suppression but also for various accident-related rescue and emergency activities and preparation for large-scale disasters and on-site response [1]. Firefighters have an increased risk of exposure to toxic gases (e.g., carbon monoxide and phosgene) and heat stress at fire sites [2]. Firefighters also suffer from high levels of job stress due to the mental tension caused by waiting time in the field and the sleep chronic deprivation caused by shift work. In fact, firefighters have higher risks of psychological problems, such as depression [3] and post-traumatic stress disorder (PTSD) [4,5,6] compared with the general population. Many firefighters suffer from sleep disorders due to exposure to shift work [7,8].

These risk factors in firefighters may also be related to neurocognitive function. Both the heat stress in a hot environment [9,10,11] and the exposure to higher stress levels [12,13] can affect neurocognitive function. Firefighters are at high-risk of PTSD [14] and depression [15,16,17], and study results indicate that these conditions are associated with cognitive impairment. In particular, results of recent studies suggest that shift work can also affect neurocognitive function [18,19,20,21]. Shift work-associated disruptions in circadian rhythms are associated with neurodegeneration [22,23]. In support of this hypothesis, one animal model experiment found that deletion of the master clock gene *Bmal1* in the mouse brain results in an increase in neuronal oxidative damage [24]. A firefighter who is engaged in long-term shift work may be at risk of cognitive decline. An experimental study performed by Rodrigues et al. found that cognitive performance in firefighters decreases after exposure to increased levels of stress [25]. However, the effects of shift work on neurocognitive function in firefighters have not yet been studied.

This study aimed to investigate the effects of shift work on neurocognitive function in firefighters by measuring and comparing cognitive functions before and after they worked the night shift.

## 2. Materials and Methods

### 2.1. Study Subjects

**Figure 1:**
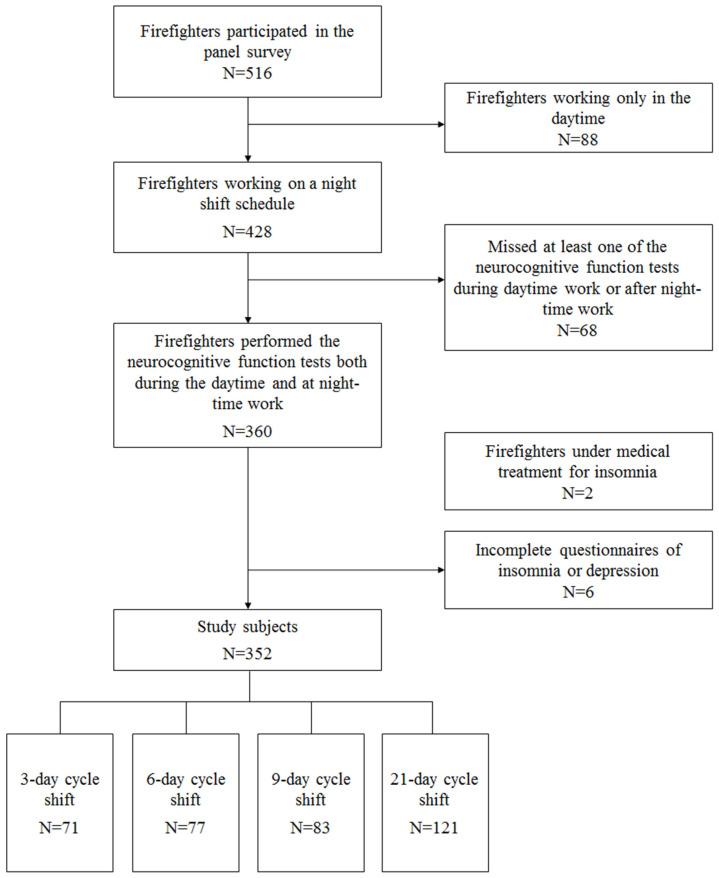
Flow chart of the study subjects.

This study was included in the SLEep Panel Study (SLEPS). The purpose of SLEPS was to investigate sleep problems among Korean firefighters. Using the two means formula for continuous variables, we calculated the sample size before conducting the SLEPS. The calculation was carried out using G*power version 3.1.9.7. When the α error was set to 0.05 and the statistical power (1–β error) to 80%, the calculated sample size was 124. In order to perform a stratified analysis in respect to sex, age group, department, job position, and shift work schedule, a sample size of 8928 was required; however, the final targeted sample size needed to be determined considering the budget constraint and time needed to construct the panel. A final target sample size of 500 was decided upon, and 516 firefighters were recruited to participate in the SLEPS. From 2017 to 2018, we conducted the questionnaire survey on a panel of 516 firefighters from eight fire stations in South Korea and performed neurocognitive function tests. Among the 516 firefighters in the panel, 88 who worked only in the daytime (i.e., no night shift work) were excluded from the analysis. We also excluded 68 firefighters who missed at least one of the neurocognitive function tests during daytime work or after nighttime work. Two firefighters being treated for insomnia by a physician were excluded because this condition could affect the results of neurocognitive function tests. Six firefighters who did not complete the insomnia or depression questionnaires were also excluded from the study. Finally, the data from 352 subjects who were currently working on a night shift schedule were included in the analysis (Figure 1).

### 2.2. Type of Shift Work

**Figure 2:**
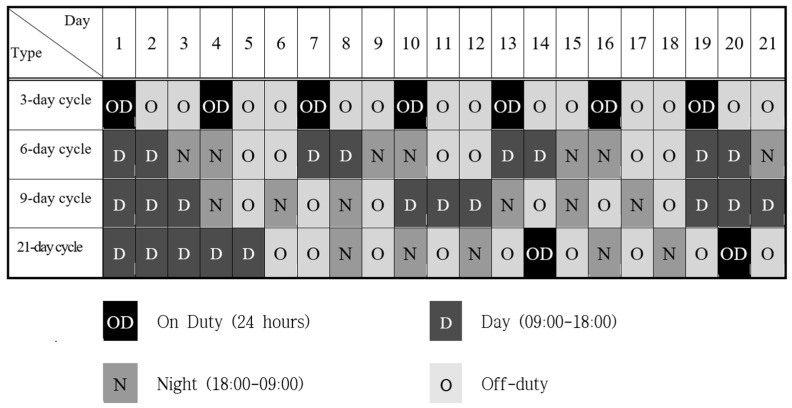
Shift work cycles of firefighters in South Korea.

Firefighters in South Korea are typically subjected to 3-, 6-, 9-, or 21-day cycle shift work. In this study, the 3-day cycle shift work schedule consisted of a full day (24 h) of work, followed by 2 days off-duty. The 6-day cycle shift work schedule consisted of 2 days of daytime work (9:00 a.m.–6:00 p.m.), followed by 2 days of nighttime work (6:00 p.m.–9:00 a.m.) and 2 days off-duty. The 9-day cycle shift work schedule consisted of 3 days of daytime work (9:00 a.m.–6:00 p.m.), followed by three consecutive sets of nighttime work and off-duty. The 21-day schedule consisted of 5 days of daytime work (9:00 a.m.–6:00 p.m.), followed in order by 2 days off-duty, three consecutive sets of nighttime work and off-duty, a full day (24 h) of work, two consecutive sets of off-duty and nighttime work, 1 day off-duty, a full day (24 h) of work, and 1 day off-duty (Figure 2).

### 2.3. Questionnaire

During the panel survey, each subject was asked to complete a self-reported questionnaire. Data on age, sex, department and job position, monthly income, education level, task, type of shift work, history of illness, family medical history, history of smoking and drinking, physical activity, and symptoms related to the target organs were collected.

### 2.4. Sleep Disorder and Depression Evaluation Tools

The Insomnia Severity Index (ISI) [26,27] was used to measure insomnia. The ISI is a self-reported questionnaire that consists of seven questions used to evaluate degrees of difficult sleep onset and sleep maintenance, satisfaction with current sleep patterns, interference with daily functioning, noticeability of impairments attributed to sleep problems, and degrees of distress or concern caused by the sleep problem [28]. Each question was scored between 0 and 4. A higher score indicated a more severe status. An ISI score ≥8 indicated the presence of a mild sleep disorder and an ISI score ≥15 indicated the presence of sleep disorder [29]. Patient Health Questionnaire-9 (PHQ-9) [30] was used to evaluate each subject’s depressive symptoms. Each item scores between 0 and 3 points for 9 depressive symptoms during the last 2 weeks. A PHQ-9 score ≥5 indicated the presence of mild depression and a score ≥10 indicated the presence of depression [31]. The Korean versions of the ISI and PHQ-9 have been validated by past studies [32,33].

### 2.5. Neurocognitive Function Testing

Neurocognitive function testing was based on the use of central nervous system vital signs (CNSVS). The CNSVS is a computerized neurocognitive test battery that was developed as a screening instrument for neurocognitive impairment; test validity and reliability have been verified [34]. CNSVS calculates standardized scores for the domains of composite memory, verbal memory, visual memory, complex attention, psychomotor speed, motor speed, processing speed, reaction time, cognitive flexibility, executive functioning, and neurocognitive index [35]. The standardized CNSVS score is calculated via a process of data standardization; the data are stratified by 10 age groups, and an average value of 100 and standard deviation of 15 are used.

To evaluate the change in scores due to shift work, the CNSVS assessments were performed during daytime work and on the next day after nighttime work. The measurement of daytime work was performed on the last day of the daytime work; it was measured as firefighters went to work. Measurement of the nighttime work was performed once on the next day after the second or third nighttime work shift, and on the next day after the full day of work. Since the measurement of the nighttime work occurred as night shift was over, both measurements of the daytime and nighttime work were taken between 9 a.m. and 10 a.m.

The CNSVS test was designed to be given within a 30-min period to increase the probability of subject compliance. To avoid the possible effects of differences between computers, all tests were performed using the same kind of computers.

### 2.6. Study Endpoints

The primary endpoint was a change in neurocognitive function after night shift; the secondary endpoint was a change in neurocognitive function after night shift according to status of depression and insomnia. The exploratory endpoint was whether there was a difference in cognitive function changes depending on the type of shift work.

### 2.7. Statistical Analysis

We performed descriptive statistical analyses for demographic and socioeconomic characteristics, and health characteristics (lifestyle habits, and status of insomnia and depression symptoms). Among the socioeconomic characteristics, monthly income was divided into high, middle, and low with 3 million KRW (2,500 USD) and 5 million KRW (4,167 USD), respectively, separating the categories. Analysis of variance (ANOVA) and chi-square tests were used to assess differences in these general characteristics between types of shift work. Paired *t*-tests were used to assess differences between the standard scores measured during daytime work and on the next day after nighttime work for each category of the CNSVS assessment. Paired *t*-tests were also performed using stratification according to the subcategories within the insomnia and depressive symptoms categories. In the stratification analysis for depressive symptoms, mild depression was included in the depression category because the number of firefighters classified as having depression was small. We used a general linear model (GLM) to analyze the association between type of shift work and the change in CNSVS score and identified whether there were differences in CNSVS score changes during daytime work and on the next day after nighttime work, according to type of shift work. To adjust for confounding variables, we used four GLM models with different variables and calculated the least square mean (LSmean) value for each type of shift work for each category of CNSVS. Model 1 did not adjust other variables. Model 2 adjusted sex, age, and educational level. Model 3 included the Model 2 adjusted variables and further adjusted the job and income variables, and the ISI/PHQ-9 scores. Multiple comparisons were also performed to determine whether the LSmean values among the types of shift work were significantly different. All analyses were performed using SAS version 9.4 software (SAS Institute, Cary, NC, USA).

### 2.8. Ethics Statement

This study was performed after obtaining approval from the Institutional Review Board (IRB) of Yonsei University Wonju Severance Christian Hospital (IRB no. CR318031).

## 3. Results

### 3.1. General Characteristics of Subjects

The 3-day cycle shift group was the oldest; a significant difference in the proportion of age groups was present across different shift groups. There were no between-group differences in sex ratios. There were statistically significant differences between the groups in socioeconomic status, such as income, highest level of education, and proportion of fire suppression work. Coffee consumption was also significantly different between groups. Health behaviors such as smoking, drinking, and exercise did not significantly differ between groups. There were no statistically significant between-group differences in mean scores of ISI and PHQ-9 scores for insomnia and depression. The ISI and PHQ-9 results indicated that there were no statistically significant differences in the proportion of insomnia and depression status among the groups of different shift work schedules (Table 1).

### 3.2. Neurocognitive Function

The results of neurocognitive function testing measured using CNSVS assessment indicated that the neurocognitive scores decreased on the next day after nighttime work, compared with during the daytime work, for all domains except reaction time and executive function. The scores for composite memory, verbal memory, visual memory, complex attention, psychomotor speed, motor speed, and neurocognitive index were significantly decreased on the next day after nighttime work (Table 2).

### 3.3. Stratifying Analysis Based on the ISI

We compared neurocognitive function during daytime work and on the next day after nighttime work by stratifying according to the ISI questionnaire results for insomnia. The composite memory, verbal memory, visual memory, and neurocognitive index in the normal group were significantly decreased on the next day after nighttime work. In the mild insomnia group, the composite memory, verbal memory, complex attention, psychomotor speed, motor speed, and neurocognitive index were significantly decreased on the day after night shift work. In the insomnia group, composite memory, verbal memory, and motor speed were significantly decreased on the next day after nighttime work. Composite memory and verbal memory were significantly decreased in all groups, regardless of insomnia status. Complex attention, psychomotor speed, and motor speed were not significantly different in the normal group but were significantly decreased on the next day after nighttime work in the mild insomnia group (Table 3).

### 3.4. Stratifying Analysis Based on the PHQ-9

We compared neurocognitive function during daytime work and on the next day after nighttime work; we stratified according to the PHQ-9 results for symptoms of depression. In both the normal group and the depression group including those with mild depressive symptoms, composite memory, verbal memory, visual memory, complex attention, psychomotor speed, motor speed, and neurocognitive index were significantly decreased on the next day after nighttime work. There was no difference in the domain in which the neurocognitive functions were significantly decreased between normal and depression group (Table 4).

### 3.5. Multivariate Analysis for the Changes of Neurocognitive Function

The GLM analysis found that in the unadjusted model, type of shift work was significantly related to the domains of verbal memory, processing speed, cognitive flexibility, and executive functioning. However, the analyses using Model 2 and Model 3, which were adjusted for confounding variables, found that type of shift work was not significantly associated with verbal memory domain. The statistical significance of all domains, except verbal memory, was maintained after adjusting for confounding variables. The composite memory, verbal memory, visual memory, complex attention, psychomotor speed, motor speed, and neurocognitive index domains, which showed statistically significant neurocognitive function decreases before and after nighttime work, were not significantly associated with type of shift work (Table 5).

Among the variables that were used as covariates other than the type of shift work, age was significantly related to neurocognitive function after nighttime work in the domain of retention time. The older firefighters had less change in neurocognitive function. The PHQ-9 score was significantly associated with neurocognitive function after nighttime work for the psychomotor speed and processing speed domains. As PHQ-9 scores increased, neurocognitive function after nighttime work was significantly more decreased. ISI score was significantly associated with changes in neurocognitive function after nighttime work for the psychomotor speed domain. However, as the ISI score increased, the change in neurocognitive function after nighttime work was significantly decreased contrary to the PHQ-9 score.

## 4. Discussion

The results of the neurocognitive function testing using the CNSVS assessment showed a significant decrease on the next day after nighttime work compared with during daytime work in the scores for six domains (composite memory, verbal memory, visual memory, complex attention, psychomotor speed, and motor speed) and the score of neurocognitive index, respectively. There was no statistically significant improvement for any domain after nighttime work.

In the multivariate analysis using GLM, the changes in neurocognitive function were associated with the type of shift work for the processing speed, cognitive flexibility, and executive functioning domains. The neurocognitive index and the domains with significant decreases after nighttime work did not show significant associations between the type of shift work and neurocognitive function change. In other words, the results confirmed that the decreases in significant neurocognitive function scores after nighttime work were the same regardless of the type of shift work.

When the data were stratified according to degree of insomnia, we found that the domains of complex attention, psychomotor speed, and motor speed score, which were not significantly decreased after nighttime work in the normal group, were significantly decreased in the mild insomnia group. Because there were differences in only some domains and there were no differences in the insomnia group except for the domain of motor speed, the generalizability of the results is limited. However, there were only 32 firefighters classified as having insomnia, so the statistical power required to identify differences was lacking. Therefore, taken together, these results suggest that even mild insomnia made the firefighters more vulnerable to the effects of nighttime work. Greater reductions in neurocognitive function were likely possible in these firefighters, compared with those without insomnia.

A firefighter’s sleep during the night shift can be disturbed by fire and emergency calls. These events may lead to sleep deprivation, which in turn can lead to poor short-term memory, slow response times, lapses in attention or concentration, and mood changes [36]. Several studies comparing the results of functional imaging of the brain for the sleep-deprived brain versus the well-rested brain have consistently found decreases in working memory in the sleep-deprived brain [37]. The results of cognitive and motor performance tests of participants with moderate sleep deprivation are affected not only by cognitive function but also by motor speed, accuracy, coordination, and attention [38]. In this study, the functions of memory domains such as composite memory, verbal memory, and visual memory, and domains in complex attention, psychomotor speed, and motor speed, were decreased after nighttime work. These changes may have been due to the sleep deprivation caused by nighttime work, resulting in a decrease in attention, and its effects on working memory, short-term memory, and motor function.

Firefighters repeatedly experience this sleep deprivation during long and repeated shift work cycles. Chronic sleep deprivation affects neurobehavioral function and shows a dose–response relationship [39]. Chronic sleep deprivation also induces changes in brain metabolism and neural activation [40]. Therefore, the deterioration in cognitive function due to the sleep deprivation of firefighters is not a one-time change; it constantly affects the brain and nervous system of firefighters and decreases the individual’s neurobehavioral function. This change increases the risk of accidents for firefighters during fire suppression activities [41] and might be a risk factor for the development of central neurological diseases such as Alzheimer’s disease in the long term [42].

In firefighters, sleep deprivation due to night work increased level of stress. A study on Dutch police officers revealed that the level of salivary cortisol was significantly increased between 4–14 months after the transition from regular day work to rotating shift work [43]. In addition, a study on German physicians reported that the level of salivary cortisol was significantly increased compared to that of who did not work night shifts [44]. Since a cortisol response is known to be associated with work stress, it can be interpreted that stress level has increased due to shift work [45,46]. Stressful conditions can induce the activation of the neuroendocrine stress system and epigenetic changes in brain, which can drive neuroplastic changes in emotional and cognitive functions [47]. Thus, it could be understood that stress induced by sleep deprivation might have influenced neurocognitive decline in firefighters after a night shift.

Previous studies of neurocognitive function changes in firefighters mainly assessed the effects of high temperatures. There is a concern that firefighters involved in fire suppression activities are exposed to high temperature environments for long periods and that the physical factors could affect neurocognitive function. Recently, an experimental study of U.S. firefighters responsible for wildland fire suppression found that there is no significant difference in the results of cognitive function assessments in very hot (45 °C) conditions compared to temperate environment (18 °C) conditions [48]. Morley et al. [49] performed a neurocognitive function test immediately after giving a heat stress similar to that of a fire suppression; they exposed the firefighters to a 50-min continuous treadmill exercise while wearing thermal protective clothing. The neurocognitive test scores did not change immediately after exercise. Our study, and these previous studies, found that firefighters in charge of fire suppression did not differ in changes in neurocognitive function test scores compared to other firefighters.

Depression, as well as sleep, can also affect a firefighter’s neurocognitive function. In previous studies, depression has been consistently associated with cognitive decline [50,51]. Firefighters also have many risk factors for depression, such as physical environment, organizational culture, and job stress [3]. Although the prevalence of depression in firefighters is not precisely known, several epidemiological studies reported high rates of depressive symptoms among firefighters [52,53]. In this study, after the other factors were adjusted for using multivariate analysis, depression was significantly related to the score changes after nighttime work for the psychomotor speed and motor speed domains. There was no difference in cognitive function before and after nighttime work between the normal and depression groups. However, only 54 firefighters were classified as having depression by PHQ-9, even if mild depression was included in the depression category. Therefore, the number of subjects was too small to confirm the difference, and further studies are needed.

In addition, measures to prevent and manage a cognitive decline due to shift work are needed. Prevention and treatment for sleep disorders are essential in reducing cognitive decline. Administrative management including the adjustment of a shift work cycle and rotating work with day shifts helps to prevent sleep disorders caused by shift work. In addition, maintenance of a sound personal sleep hygiene is also crucial at the individual level. Non-pharmacological therapies are also useful, especially for firefighters with sleep disorders. It has been reported that melatonin improved sleep efficiency [54,55]; taking a melatonin supplement or eating foods rich in melatonin might assist sleeping in firefighters on a night shift. In addition, cognitive behavioral therapy could improve sleep quality in firefighters with sleep disorders. A proper use of these non-pharmacological therapies will help to prevent cognitive decline due to sleep deprivation [56,57,58].

This study had several limitations. The CNSVS testing was conducted directly during the daytime and immediately after the night shift, but most of the tests were performed only once or twice. It was not followed up longitudinally. In addition, the specific firefighting activities at the nighttime fire scenes, which occurred before measurements were taken, are important factors that affect cognitive function, but no detailed records and observations of these activities were available. These activities likely acted as unmeasured confounders and resulted in bias. Furthermore, although this study included firefighters involved in various occupations and from many fire stations, the numbers of participants available for the stratified analyses were relatively small. The statistical power was low when the effects of stratification were analyzed according to PHQ-9 category because only a small number of firefighters was classified as having depression. To overcome these limitations, a longitudinal follow-up study involving more participants is needed in the future.

## 5. Conclusions

We compared neurocognitive function using CNSVS testing before and after nighttime work performed by firefighters in South Korea who were working in shifts. We found that after nighttime work, neurocognitive functions were generally lower, compared with those during daytime work. In particular, neurocognitive function was decreased for the memory and attention domains; and psychomotor and motor function were also decreased. This result is consistent with the effects of sleep deprivation found by previous studies. Therefore, the results of this study suggested that working the night shift in firefighters is a risk factor for neurocognitive decline.

## Figures and Tables

**Table 1:** General characteristics of the subjects.

Characteristics	Shift Work Schedule				All Subjects *N* = 352	*p*-Value
	3-Day Cycle *N* = 71	6-Day Cycle *N* = 77	9-Day Cycle *N* = 83	21-Day Cycle *N* = 121		
Age (years)						0.043 ^3–6^
20–29	4 (5.6%)	11 (14.3%)	11 (13.3%)	14 (11.6%)	40 (11.4%)	
30–39	21 (29.6%)	35 (45.5%)	29 (34.9%)	47 (38.8%)	132 (37.5%)	
40–49	30 (42.3%)	19 (24.7%)	24 (28.9%)	48 (39.7%)	121 (34.4%)	
50–59	16 (22.5%)	12 (15.6%)	19 (22.9%)	12 (9.9%)	59 (16.8%)	
Mean ± SD	42.5 ± 8.1	38.4 ± 9.3	41.2 ± 9.0	39.1 ± 8.0	40.1 ± 8.7	0.012 ^3–6,3–21^
Sex						0.051
Men	61 (85.9%)	73 (94.8%)	80 (96.4%)	114 (94.2%)	328 (93.2%)	
Women	10 (14.1%)	4 (5.2%)	3 (3.6%)	7 (5.8%)	24 (6.8%)	
Job						0.009 ^3–21,6–21^
Fire suppression	14 (19.7%)	21 (27.3%)	25 (30.1%)	51 (42.1%)	111 (31.5%)	
Others	57 (80.3%)	56 (72.7%)	58 (69.9%)	70 (57.9%)	241 (68.5%)	
Education						0.046 ^3–6,6–21^
High school	10 (14.1%)	24 (31.2%)	18 (21.7%)	18 (14.9%)	70 (19.9%)	
2-year degree	23 (32.4%)	23 (29.9%)	18 (21.7%)	36 (29.7%)	100 (28.4%)	
4-year degree	38 (53.5%)	30 (39.0%)	47 (56.6%)	67 (55.4%)	182 (51.7%)	
Monthly income						<0.001 ^3–6,3–21,6–9,9–21^
Missing	0	0	2	0	2	
Low	11 (15.5%)	33 (42.9%)	12 (14.8%)	37 (30.6%)	93 (26.6%)	
Middle	32 (45.1%)	32 (41.6%)	42 (51.9%)	62 (51.2%)	168 (48.0%)	
High	28 (39.4%)	12 (15.6%)	27 (33.3%)	22 (18.2%)	89 (25.4%)	
Smoking						0.576
Missing	0	0	0	1	1	
Never	29 (40.8%)	37 (48.0%)	33 (39.8%)	40 (33.3%)	139 (39.6%)	
Past smoker	24 (33.8%)	22 (28.6%)	21 (25.3%)	45 (37.5%)	112 (31.9%)	
Current light smoker	10 (14.1%)	10 (13.0%)	17 (20.5%)	21 (17.5%)	58 (16.5%)	
Current heavy smoker	8 (11.3%)	8 (10.4%)	12 (14.5%)	14 (11.7%)	42 (12.0%)	
Alcohol						0.996
Missing	2	3	1	2	8	
No	15 (21.7%)	17 (23.0%)	16 (19.5%)	24 (20.2%)	72 (20.9%)	
Normal drinking	41 (59.4%)	41 (55.4%)	48 (58.5%)	69 (58.0%)	199 (57.9%)	
Heavy drinking	13 (18.8%)	16 (21.6%)	18 (22.0%)	26 (21.8%)	73 (21.2%)	
Regular exercise						0.636
No	33 (46.5%)	36 (46.8%)	44 (53.0%)	53 (43.8%)	166 (47.2%)	
Yes	38 (53.5%)	41 (53.2%)	39 (47.0%)	68 (56.2%)	186 (52.8%)	
Caffeine						0.045 ^3–6,3–9^
Missing	5	7	2	4	18	
No	8 (12.1%)	16 (22.9%)	15 (18.5%)	18 (15.4%)	57 (17.1%)	
Light coffee drinking	25 (37.9%)	35 (50.0%)	45 (55.6%)	61 (52.1%)	166 (49.7%)	
Moderate to heavy coffee drinking	33 (50.0%)	19 (27.1%)	21 (25.9%)	38 (32.5%)	111 (33.2%)	
Insomnia						0.148
Normal (≤ 7)	43 (60.6%)	31 (40.3%)	44 (53.0%)	68 (56.2%)	186 (52.8%)	
Mild Insomnia (8–14)	21 (29.6%)	40 (51.9%)	32 (38.6%)	41 (33.9%)	134 (38.1%)	
Insomnia (≥ 15)	7 (9.9%)	6 (7.8%)	7 (8.4%)	12 (9.9%)	32 (9.1%)	
Mean ± SD	7.3 ± 2.2	8.8 ± 2.4	7.7 ± 4.7	7.2 ± 5.3	7.7 ± 5.0	0.151
Depression						0.896
Normal (≤ 4)	60 (84.5%)	64 (83.1%)	71 (85.5%)	103 (85.1%)	298 (84.7%)	
Mild depression (5–9)	9 (12.7%)	9 (11.7%)	11 (13.3%)	14 (11.6%)	43 (12.2%)	
Depression (≥ 10)	2 (2.8%)	4 (5.2%)	1 (1.2%)	4 (3.3%)	11 (3.1%)	
Mean ± SD	2.2 ± 2.8	2.4 ± 3.1	2.1 ± 2.3	2.0 ± 2.9	2.1 ± 2.8	0.771

Values are presented as means ± standard deviation (SD) or number (%). Analyzed by ANOVA or chi-square test. Types of shift work with significant differences in multiple comparisons are listed with a superscript (^3^: 3-day cycle, ^6^: 6-day cycle, ^9^: 9-day cycle, ^21^: 21-day cycle). Heavy smoker: smoking of ≥15 cigarettes per day. Heavy drinking: consumption of ≥7 glasses per day at least two times per week in men, ≥5 glasses per day at least two times per week in women. Moderate to heavy coffee drinking: consumption of ≥3 cups per day (moderate: 3–4 cups, heavy: ≥5 cups). Regular exercise: exercise above moderate intensity for more than 1 hour a week.

**Table 2:** Comparison of the neurocognitive function during the daytime work and after the night shift work of firefighters.

Domain	Mean ± SD (*N* = 352)		*p*-Value
	During Day Work	After Night Work	
Composite memory	90.6 ± 19.1	84.7 ± 19.7	<0.001
Verbal memory	87.7 ± 20.0	81.3 ± 21.9	<0.001
Visual memory	97.1 ± 16.3	94.0 ± 16.6	0.001
Complex attention	97.8 ± 18.2	93.3 ± 32.4	0.007
Psychomotor speed	112.4 ± 15.4	110.1 ± 15.2	<0.001
Motor speed	111.0 ± 15.1	108.7 ± 14.1	<0.001
Processing speed	107.6 ± 15.7	107.4 ± 17.1	0.860
Reaction time	92.4 ± 15.0	92.7 ± 17.1	0.711
Cognitive flexibility	106.2 ± 16.9	105.8 ± 19.1	0.671
Executive functioning	107.0 ± 16.5	107.2 ± 18.5	0.799
Neurocognitive index	99.9 ± 11.6	97.4 ± 13.4	<0.001

All subgroups were analyzed by paired *t*-test.

**Table 3:** Comparison of the neurocognitive function during the daytime work and after the night shift work of firefighters according to the Insomnia Severity Index (ISI) category.

Domain	ISI Category	*N*	Mean ± SD (*n* = 352)		*p*-Value
			During Daytime Work	Post Nighttime Work	
Composite memory	Normal	186	90.2 ± 20.1	84.8 ± 19.5	<0.001
	Mild insomnia	134	90.6 ± 17.2	85.4 ± 19.4	0.002
	Insomnia	32	92.8 ± 21.6	81.5 ± 21.9	0.012
Verbal memory	Normal	186	87.7 ± 20.6	82.7 ± 22.5	<0.001
	Mild insomnia	134	87.4 ± 19.1	80.4 ± 20.3	<0.001
	Insomnia	32	89.7 ± 20.1	77.2 ± 24.6	0.001
Visual memory	Normal	186	96.6 ± 16.8	92.8 ± 15.8	0.006
	Mild insomnia	134	97.6 ± 14.9	95.8 ± 17.1	0.269
	Insomnia	32	98.3 ± 18.9	92.8 ± 18.6	0.110
Complex attention	Normal	186	96.1 ± 20.3	94.3 ± 23.7	0.260
	Mild insomnia	134	100.1 ± 15.0	92.2 ± 43.3	0.027
	Insomnia	32	98.2 ± 17.0	92.7 ± 22.4	0.227
Psychomotor speed	Normal	186	111.7 ± 16.5	110.2 ± 15.9	0.056
	Mild insomnia	134	114.1 ± 14.5	111.5 ± 14.5	0.008
	Insomnia	32	109.3 ± 11.3	104.3 ± 13.5	0.069
Motor speed	Normal	186	110.1 ± 15.8	108.6 ± 14.7	0.053
	Mild insomnia	134	112.2 ± 14.8	108.3 ± 13.5	0.001
	Insomnia	32	111.4 ± 12.1	104.8 ± 13.6	0.007
Processing speed	Normal	186	107.7 ± 15.9	106.5 ± 16.9	0.298
	Mild insomnia	134	109.1 ± 15.9	110.2 ± 17.4	0.341
	Insomnia	32	100.8 ± 11.3	101.4 ± 15.0	0.845
Reaction time	Normal	186	92.4 ± 15.2	92.3 ± 16.8	0.931
	Mild insomnia	134	93.5 ± 15.2	93.3 ± 17.3	0.872
	Insomnia	32	88.5 ± 12.3	93.0 ± 19.2	0.190
Cognitive flexibility	Normal	186	104.8 ± 18.8	105.1 ± 18.9	0.809
	Mild insomnia	134	108.4 ± 14.0	106.8 ± 20.1	0.306
	Insomnia	32	105.3 ± 15.4	106.0 ± 15.8	0.821
Executive functioning	Normal	186	105.6 ± 18.3	106.6 ± 18.3	0.426
	Mild insomnia	134	109.2 ± 13.8	108.0 ± 19.8	0.414
	Insomnia	32	105.8 ± 15.2	108.0 ± 14.7	0.397
Neurocognitive index	Normal	186	99.0 ± 13.0	97.3 ± 12.7	0.009
	Mild insomnia	134	101.4 ± 9.6	97.9 ± 14.9	0.001
	Insomnia	32	98.9 ± 10.9	95.4 ± 10.3	0.089

All subgroups were analyzed by paired *t*-test.

**Table 4:** Comparison of the neurocognitive function during the daytime work and after the night shift work of firefighters according to the Patient Health Questionnaire-9 (PHQ-9) category.

Domain	PHQ-9 Category	*N*	Mean ± SD (*n* = 352)		*p*-Value
			During Daytime Work	Post Nighttime Work	
Composite memory	Normal	298	90.1 ± 19.1	84.6 ± 19.7	<0.001
	Depression	54	93.3 ± 19.4	85.3 ± 19.7	0.003
Verbal memory	Normal	298	87.3 ± 19.8	81.1 ± 22.0	<0.001
	Depression	54	90.1 ± 20.9	82.7 ± 21.3	<0.001
Visual memory	Normal	298	96.8 ± 16.3	94.0 ± 16.3	0.010
	Depression	54	99.1 ± 16.4	93.8 ± 18.4	0.040
Complex attention	Normal	298	97.2 ± 19.0	93.1 ± 34.0	0.030
	Depression	54	101.0 ± 12.7	94.5 ± 22.1	0.017
Psychomotor speed	Normal	298	112.3 ± 15.8	111.0 ± 15.0	0.035
	Depression	54	112.9 ± 13.5	105.4 ± 15.9	<0.001
Motor speed	Normal	298	110.6 ± 15.5	108.6 ± 14.1	0.002
	Depression	54	113.2 ± 12.7	105.8 ± 14.3	<0.001
Processing speed	Normal	298	108.1 ± 15.9	108.6 ± 16.9	0.626
	Depression	54	104.5 ± 14.2	101.2 ± 16.8	0.143
Reaction time	Normal	298	92.7 ± 15.1	92.5 ± 17.3	0.808
	Depression	54	90.8 ± 14.5	93.7 ± 16.4	0.142
Cognitive flexibility	Normal	298	106.0 ± 17.4	105.9 ± 19.0	0.921
	Depression	54	107.6 ± 14.1	105.6 ± 19.7	0.471
Executive functioning	Normal	298	106.8 ± 16.9	107.4 ± 18.4	0.545
	Depression	54	108.2 ± 13.7	106.6 ± 19.4	0.544
Neurocognitive index	Normal	298	99.7 ± 12.0	97.4 ± 13.6	<0.001
	Depression	54	101.1 ± 9.4	96.9 ± 12.1	0.003

All subgroups were analyzed by paired *t*-test.

**Table 5:** Comparison of the changes of neurocognitive function according to the type of shift work.

Domain	Type of Shift Work	Model 1		Model 2		Model 3	
		LSmeans	*p*-Value	LSmeans	*p*-Value	LSmeans	*p*-Value
Composite memory	3-day cycle	8.38	0.503	9.26	0.493	8.68	0.483
	6-day cycle	4.74		5.06		4.59	
	9-day cycle	3.81		4.82		4.18	
	21-day cycle	6.50		7.35		7.07	
Verbal memory	3-day cycle	−20.17	0.030	−15.78	0.060	−15.92	0.071
	6-day cycle	−15.49 ^9^		−12.13		−12.07	
	9-day cycle	−25.95 ^6,21^		−13.01		−21.21	
	21-day cycle	−17.19 ^9^		−15.78		−12.68	
Visual memory	3-day cycle	6.20	0.327	6.39	0.246	5.34	0.313
	6-day cycle	1.30		0.61		0.12	
	9-day cycle	1.49		1.30		0.69	
	21-day cycle	3.79		3.59		3.29	
Complex attention	3-day cycle	0.21	0.563	0.29	0.499	−0.15	0.547
	6-day cycle	7.03		7.75		6.86	
	9-day cycle	4.11		4.53		4.29	
	21-day cycle	5.63		6.50		6.32	
Psychomotor speed ^†^	3-day cycle	2.04	0.307	2.40	0.215	1.81	0.279
	6-day cycle	0.31		0.10		−0.41	
	9-day cycle	3.66		3.96		3.15	
	21-day cycle	2.71		2.68		2.15	
Motor speed ^‡^	3-day cycle	4.21	0.131	3.25	0.075	3.20	0.112
	6-day cycle	3.14		1.92		1.69	
	9-day cycle	4.31		3.29		2.81	
	21-day cycle	1.00		−0.39		−0.50	
Processing speed ^††^	3-day cycle	−2.66 ^21^	<0.001	−0.23 ^21^	<0.001	−1.47 ^21^	<0.001
	6-day cycle	−4.21 ^9,21^		−2.37 ^9,21^		−2.93 ^9,21^	
	9-day cycle	1.12 ^6^		3.60 ^6^		2.64 ^6^	
	21-day cycle	3.88 ^3,6^		6.36 ^3,6^		5.50 ^3,6^	
Reaction time	3-day cycle	−0.69	0.986	1.64	0.907	2.52	0.860
	6-day cycle	−0.56		0.71		1.47	
	9-day cycle	0.04		2.41		3.50	
	21-day cycle	−0.09		1.57		2.41	
Cognitive flexibility	3-day cycle	−3.24 ^21^	<0.001	−3.02 ^21^	<0.001	−2.88 ^21^	<0.001
	6-day cycle	−3.74 ^21^		−4.53 ^21^		−4.62 ^21^	
	9-day cycle	0.20 ^21^		−0.36 ^21^		−0.05 ^21^	
	21-day cycle	5.25 ^3,6,9^		4.90 ^3,6,9^		5.18 ^3,6,9^	
Executive functioning	3-day cycle	−3.52 ^21^	<0.001	−3.21 ^21^	<0.001	−3.10 ^21^	<0.001
	6-day cycle	−4.78 ^21^		−5.51 ^21^		−5.39 ^21^	
	9-day cycle	−0.46 ^21^		−0.83 ^21^		−0.59 ^21^	
	21-day cycle	4.77 ^3,6,9^		4.42 ^3,6,9^		4.80 ^3,6,9^	
Neurocognitive index	3-day cycle	1.46	0.291	2.19	0.269	1.52	0.225
	6-day cycle	1.57		1.77		2.92	
	9-day cycle	2.35		3.00		4.55	
	21-day cycle	3.99		4.53		2.07	

Analyzed by general linear model. Types of shift work with significant differences in multiple comparisons are listed with a superscript (^3^: 3-day cycle, ^6^: 6-day cycle, ^9^: 9-day cycle, ^21^: 21-day cycle). Model 1: unadjusted. Model 2: adjustment for age, sex, and education level. Model 3: Model 2 + adjustments for income, job, and ISI/PHQ-9 scores. LSmeans: least square means. ^†^ PHQ-9 score (β: 1.05, 95% confidence interval (CI) 0.54 to 1.56) was significantly associated with change in the domain of psychomotor speed. ^‡^ Age was significantly associated with change in the domain of reaction time in Model 2 (β: −0.30, 95% CI −0.48 to −0.12) and Model 3 (β: −0.26, 95% CI −0.48 to −0.03). ^††^ ISI (β: −0.58, 95% CI −0.94 to −0.21) and PHQ-9 (β: 1.32, 95% CI 0.67 to −1.98) scores were significantly associated with change in the domain of processing speed.
